# Supplementary material for: Small Cajal Body-Specific RNA12 Promotes Carcinogenesis through Modulating Extracellular Matrix Signaling in Bladder Cancer
Source: Cancers (Basel). 2024 Jan 23;16(3):483. doi: 10.3390/cancers16030483 (PMC10854576; doi:10.3390/cancers16030483)
Supplement: Supplementary file 1 [file cancers-16-00483-s001.zip › cancers-2783696-supplementary.pdf]

Supplementary Materials: Small Cajal Body-Specific RNA12 Promotes Carcinogenesis through Modulating Extracellular Matrix Signaling in Bladder Cancer

Qinchen Lu, Jiandong Wang, Yuting Tao, Jialing Zhong, Zhao Zhang, Chao Feng, Xi Wang, Tianyu Li, Rongquan He, Qiuyan Wang and Yuanliang Xie

Table S1. The clinical characteristics of 52 BLCA patients.

Characteristics	Number of patients
Gender	
Male	44
Female	8
Age	
<55	11
≥55	39
TNM stage	
I, II	34
III,IV	16
Smoking	
Yes	24
No	26
Grade	
High	41
Low	7
Lymphatic metastasis	
Yes	13
No	29

Table S2. ISH scores of SCARNA12 in clinical tissue samples.

Tissue type	Cases	SCARNA12 expression level		P value
		High level	Low level	
Tumour tissues	140	118 (84.3%)	22 (15.7%)	<0.001
Adjacent tissues	51	11 (21.6%)	40 (78.4%)	

Table S3. The panel of antibodies used for CyTOF.

Antibodies	Clone	Source	Product_ID	Label
ALDH	44	Fluidigm	3147015B	147Sm
Androgen Receptor	G122-434	Fluidigm	3154018B	154Sm
c-Myc	9E10	Fluidigm	3176012B	176Yb
CK6	EPR4515	Abcam	ab222395	146Nd
CK5	CK5	Abcam	ab53121	164Dy
Estrogen Receptor -α	C-542	Abcam	ab66102	142Nd

Estrogen Receptor-β	14C8	Abcam	ab288	174Yb
Ki67	Ki67	BioLegend	350523	161Dy
KLF4	D1F2	Fluidigm	3162022A	162Dy
LGR5	SA222C5	BioLegend	373802	155Gd
MET	D1C2	Fluidigm	3167017A	167Er
MUC1	SM3	Abcam	ab22711	168Er
Nanog	N31-355	Fluidigm	3169014A	169Tm
Notch2	MHN2-25	Fluidigm	3165026B	165Ho
OV6	OV-6	R&D	MAB2020	152Sm
p21	12D1	Fluidigm	3159026A	159Tb
p53	7F5	Fluidigm	3143018A	143Nd
Sox2	O30-678	Fluidigm	3150019B	150Nd
Vimentin	RV202	Fluidigm	3156023A	156Gd
CD45	HI30	BioLegend	304045	Y89
CD13	WM15	Fluidigm	3160014B	160Gd
CD24	ML5	Fluidigm	3166007B	166Er
CD34	581	Fluidigm	3149013B	149Sm
CD44	IM7	Fluidigm	3171003B	171Yb
CD47	CC2C6	Fluidigm	3209004B	209Bi
CD54	HA58	Fluidigm	3170014B	170Er
CD90	5E10	BioLegend	328102	158Gd
CD104	58XB4	Fluidigm	3173008B	173Yb
CD133	170411	R&D	MAB11331	153Eu
CD166	3A6	BioLegend	343902	145Nd
CD274 (PD-L1)	29E.2A3	Fluidigm	3175017B	175Lu
CD326 (EpCAM)	9C4	Fluidigm	3141006B	141Pr
CD333 (FGFR3)	136334	R&D	MAB766	151Eu

Table S4. Detection of SCARNA12 interacting protein using Chromatin Isolation by RNA Purification (ChIRP) .

Accession	Score	Mass	Matches	Sequences	emPAI	Protein description
P02760	10909	39886	257 (218)	2 (2)	0.49	Protein AMBP OS=Homo sapiens OX=9606 GN=AMBP PE=1 SV=1
P41222	4167	21243	151 (119)	3 (3)	0.8	Prostaglandin-H2 D-isomerase OS=Homo sapiens OX=9606 GN=PTGDS PE=1 SV=1
P04264	710	66170	29 (22)	23 (19)	1.77	Keratin, type II cytoskeletal 1 OS=Homo sapiens OX=9606 GN=KRT1 PE=1 SV=6
P35908	337	65678	16 (10)	15 (10)	0.63	Keratin, type II cytoskeletal 2 epidermal OS=Homo sapiens OX=9606 GN=KRT2 PE=1 SV=2
P13647	145	62568	7 (5)	7 (5)	0.29	Keratin, type II cytoskeletal 5 OS=Homo sapiens OX=9606 GN=KRT5 PE=1 SV=3
P05787	144	53671	3 (3)	2 (2)	0.13	Keratin, type II cytoskeletal 8 OS=Homo sapiens OX=9606 GN=KRT8 PE=1 SV=7
P02538	96	60293	5 (3)	5 (3)	0.17	Keratin, type II cytoskeletal 6A OS=Homo sapiens OX=9606 GN=KRT6A PE=1 SV=3
P13645	673	59020	26 (24)	22 (21)	2.48	Keratin, type I cytoskeletal 10 OS=Homo sapiens OX=9606 GN=KRT10 PE=1 SV=6
P35527	251	62255	17 (11)	14 (10)	0.76	Keratin, type I cytoskeletal 9 OS=Homo sapiens OX=9606 GN=KRT9 PE=1 SV=3
P08779	155	51578	6 (6)	6 (6)	0.45	Keratin, type I cytoskeletal 16 OS=Homo sapiens OX=9606 GN=KRT16 PE=1 SV=4
P05783	61	48029	3 (2)	3 (2)	0.14	Keratin, type I cytoskeletal 18 OS=Homo sapiens OX=9606 GN=KRT18 PE=1 SV=2
P02753	638	23337	35 (23)	1 (1)	0.14	Retinol-binding protein 4 OS=Homo sapiens OX=9606 GN=RBP4 PE=1 SV=3
P02768	186	71317	12 (6)	8 (3)	0.2	Serum albumin OS=Homo sapiens OX=9606 GN=ALB PE=1 SV=2
P35579	160	227646	11 (4)	11 (4)	0.06	Myosin-9 OS=Homo sapiens OX=9606 GN=MYH9 PE=1 SV=4
P01834	154	11929	5 (3)	2 (2)	1.14	Immunoglobulin kappa constant OS=Homo sapiens OX=9606 GN=IGKC PE=1 SV=2
P60709	126	42052	12 (7)	9 (6)	0.7	Actin, cytoplasmic 1 OS=Homo sapiens OX=9606 GN=ACTB PE=1 SV=1
A0A0C4DH25	122	12621	1 (1)	1 (1)	0.27	Immunoglobulin kappa variable 3D-20 OS=Homo sapiens OX=9606 GN=IGKV3D-20 PE=3 SV=1
P02671	87	95656	1 (1)	1 (1)	0.03	Fibrinogen alpha chain OS=Homo sapiens OX=9606 GN=FGA PE=1 SV=2
Q8WVN6	82	27307	1 (1)	1 (1)	0.12	Secreted and transmembrane protein 1 OS=Homo sapiens OX=9606 GN=SECTM1 PE=1 SV=2
P0C0L4	74	194261	1 (1)	1 (1)	0.02	Complement C4-A OS=Homo sapiens OX=9606 GN=C4A PE=1 SV=2
P68871	70	16102	2 (2)	2 (2)	0.47	Hemoglobin subunit beta OS=Homo sapiens OX=9606 GN=HBB PE=1 SV=2
P62805	69	11360	3 (3)	2 (2)	1.22	Histone H4 OS=Homo sapiens OX=9606 GN=HIST1H4A PE=1 SV=2
Q9BQE3	65	50548	1 (1)	1 (1)	0.07	Tubulin alpha-1C chain OS=Homo sapiens OX=9606 GN=TUBA1C PE=1 SV=1
O00187	62	77193	1 (1)	1 (1)	0.04	Mannan-binding lectin serine protease 2 OS=Homo sapiens OX=9606 GN=MASP2 PE=1 SV=4
O43390	46	71184	1 (1)	1 (1)	0.05	Heterogeneous nuclear ribonucleoprotein R OS=Homo sapiens OX=9606 GN=HNRNPR PE=1 SV=1
P69905	41	15305	1 (1)	1 (1)	0.22	Hemoglobin subunit alpha OS=Homo sapiens OX=9606 GN=HBA1 PE=1 SV=2
P0C0S5	40	13545	1 (1)	1 (1)	0.25	Histone H2A.Z OS=Homo sapiens OX=9606 GN=H2AFZ PE=1 SV=2
P19338	38	76625	2 (1)	2 (1)	0.04	Nucleolin OS=Homo sapiens OX=9606 GN=NCL PE=1 SV=3
Q8TF72	37	218321	1 (1)	1 (1)	0.01	Protein Shroom3 OS=Homo sapiens OX=9606 GN=SHROOM3 PE=1 SV=2
P16402	37	22336	15 (2)	3 (2)	0.32	Histone H1.3 OS=Homo sapiens OX=9606 GN=HIST1H1D PE=1 SV=2
P07911	36	72451	1 (1)	1 (1)	0.05	Uromodulin OS=Homo sapiens OX=9606 GN=UMOD PE=1 SV=1
P20929	31	775393	5 (1)	5 (1)		Nebulin OS=Homo sapiens OX=9606 GN=NEB PE=1 SV=5
O15018	30	303964	3 (1)	3 (1)	0.01	PDZ domain-containing protein 2 OS=Homo sapiens OX=9606 GN=PDZD2 PE=1 SV=4
Q6NXT2	28	15318	3 (1)	3 (1)	0.22	Histone H3.3C OS=Homo sapiens OX=9606 GN=H3F3C PE=1 SV=3
Q86Y46	27	59457	2 (1)	2 (1)	0.06	Keratin, type II cytoskeletal 73 OS=Homo sapiens OX=9606 GN=KRT73 PE=1 SV=1

P62979	27	18296	3 (1)	3 (1)	0.18	Ubiquitin-40S ribosomal protein S27a OS=Homo sapiens OX=9606 GN=RPS27A PE=1 SV=2
Q14BN4	25	95995	1 (1)	1 (1)	0.03	Sarcolemmal membrane-associated protein OS=Homo sapiens OX=9606 GN=SLMAP PE=1 SV=1
Q5VTE0	25	50495	1 (1)	1 (1)	0.07	Putative elongation factor 1-alpha-like 3 OS=Homo sapiens OX=9606 GN=EEF1A1P5 PE=5 SV=1
Q8N448	23	77154	5 (1)	1 (1)	0.04	Ligand of Numb protein X 2 OS=Homo sapiens OX=9606 GN=LNX2 PE=1 SV=1
Q8IX30	22	114399	2 (1)	2 (1)	0.03	Signal peptide, CUB and EGF-like domain-containing protein 3 OS=Homo sapiens OX=9606 GN=SCUBE3 PE=1
Q8N3F8	21	94352	1 (1)	1 (1)	0.03	MICAL-like protein 1 OS=Homo sapiens OX=9606 GN=MICALL1 PE=1 SV=2
Q8WZ42	19	3842904	14 (1)	13 (1)		Titin OS=Homo sapiens OX=9606 GN=TTN PE=1 SV=4
P98088	18	601963	2 (1)	2 (1)	0.01	Mucin-5AC OS=Homo sapiens OX=9606 GN=MUC5AC PE=1 SV=4
P04198	16	49930	3 (1)	1 (1)	0.07	N-myc proto-oncogene protein OS=Homo sapiens OX=9606 GN=MYCN PE=1 SV=2
